# Mechanisms of P-Glycoprotein Regulation Under Exogenous and Endogenous Oxidative Stress In Vitro

**DOI:** 10.32607/actanaturae.11759

**Published:** 2022

**Authors:** Yu. V. Abalenikhina, A. V. Shchulkin, P. Yu. Mylnikov, E. D. Rokunov, E. N. Yakusheva

**Affiliations:** Ryazan State Medical University named after Academician I.P. Pavlov, Ryazan, 390026 Russia

**Keywords:** P-glycoprotein, oxidative stress, Western blot, Nrf2, HIF1α, CAR, PXR, Caco-2 cell line

## Abstract

We investigated the mechanisms of P-glycoprotein (P-gp) transporter regulation in Caco-2 cells under exogenous and endogenous oxidative stress (OS). Exogenous OS was modeled by exposure of the growth medium to hydrogen peroxide at concentrations of 0.1, 0.5, and 1 μM for 24 h or 10 μM for 72 h. Endogenous OS was modeled by incubating cells with DL-buthionine sulfoximine (BSO, gamma-glutamylcysteine synthetase inhibitor) at a concentration of 10, 50, and 100 μM for 24 h. The levels of intracellular reactive oxygen species (ROS) were assessed using MitoTracker Red CM-H2XRos fluorescent probes. Relative P-gp contents were analyzed using Western blot. Exogenous and endogenous OS was shown to increase relative to P-gp contents. An important role played by the Nrf2-Keap1 signaling pathway in increasing the P-gp contents under H_2_O_2_-induced exogenous OS was revealed using specific inhibitors. The transcription factor HIF1 is involved in the regulation of the P-gp levels under 24-hour exogenous OS, and the transcription factor CAR is involved in the regulation of transporter levels under 72-hour OS. All tested transcription factors and signaling pathways are involved in P-gp induction under endogenous OS. Most likely, this is associated with the bimodal effect of BSO on Pgp. On the one hand, BSO induces the development of OS; on the other, BSO, as a xenobiotic, is able to stimulate PXR and CAR, which, in turn, increase the P-gp contents.

## INTRODUCTION

P-glycoprotein (P-gp, ABCB1), a product of the multidrug resistance gene (MDR1), is an ATP-dependent transporter protein localized on the cytoplasmic membranes of intestinal enterocytes, hepatocytes, renal tubule epithelial cells, and blood–tissue barrier endothelial cells [1].

P-gp displays wide substrate specificity and acts as an efflux transporter that controls the cell’s uptake of transporter substrates, such as antitumor, antihypertensive, and antihistamine drugs, cardiac glycosides, antiplatelet agents, anticoagulants, steroid and thyroid hormones, antibiotics, HIV proteinase inhibitors, and immunosuppressants. Given these properties, P-gp is believed to play an important role in the protection of tumor cells from cytotoxic agents (development of multidrug tumor resistance), inhibition of substrate transport into fetal tissues and sequestered organs (brain, testicles), and the pharmacokinetics (absorption, distribution, excretion) of drugs [2, 3].

A number of substances and factors can affect P-gp activity and expression. For example, P-gp expression changes occur in the blood-brain barrier in neurological diseases (epilepsy) [4] and in gastric cancer and osteosarcoma cells [5, 6].

Oxidative stress (OS) is a typical pathological process induced by a shift in the balance between oxidants and antioxidants towards oxidants, which leads to redox signaling and control impairment and/or biomacromolecule damage [7]. OS plays an important role in the pathogenesis of many diseases, including those whose character is cardiovascular, oncological, bronchopulmonary, ophthalmic, etc. [8]. Incubation of a rat hepatocyte culture with H_2_O_2_ (0.5–1 mM, 72 h) was shown to increase the expression of the P-gp gene and the level and activity of the transporter protein encoded by this gene [9]. Exposure of a primary rat endothelial cell culture to H_2_O_2_ at a concentration of up to 500 μM for 48 h was found to increase P-gp expression and, to a lesser extent, affect the activity of the transporter protein [10]. At the same time, treatment of hCMEC/D3 cells (an in vitro blood–brain barrier model) with H_2_O_2_ (0.5–5 mM, 20 min) reduced the transport activity of P-gp in [11]. Exposure of endothelial rat brain cells to H_2_O_2_ at a concentration of 200 μM was shown to cause the development of OS, increase the expression of mRNAs of the mdr1a and mdr1b genes encoding P-gp, as well as elevate the synthesis of the P-gp protein. Pretreatment of cells with polyethylene glycol-catalase reversed these changes [12]. Exposure of rat hepatocytes to a catalase inhibitor, 3-amino-1,2,4-triazole (2–4 mM for 72 h or 10 mM for 1 h), led to increased expression of mdr1b mRNA and P-gp [9]. On the contrary, antioxidants (1 mM ascorbate, 10 mM mannitol) considerably suppressed mdr1b mRNA expression and P-gp overexpression [13, 14]. Caco-2 cells cultured in a medium containing 1 μM/L H_2_O_2_ increased P-gp expression, while H_2_O_2_ at a concentration of 10 mM/L decreased the expression of the transporter [15]. P-gp expression in mitochondria of D407 cells (retinal pigment epithelium) was increased by H_2_O_2_ and suppressed by antioxidants [16].

In our laboratory, studies on Caco-2 cells showed that short-term (3 h) exposure to H_2_O_2_ at concentrations of 10 and 50 μM decreases P-gp activity and at 100 μM also reduces the levels of the transporter protein. Increasing the exposure duration to 24 and 72 h revealed P-gp induction at low H_2_O_2_ concentrations (0.1–1 μM, 24 h and 10 μM, 72 h), and increasing the H_2_O_2_ concentration to 100 μM and higher led to a decrease in the P-gp content and activity [17].

Thus, most studies have demonstrated that pro-oxidants increase P-gp expression and activity, which can be suppressed during the adaptation process failure and decompensated OS development.

A decrease in P-gp levels under OS conditions is believed to be associated with damage to the transporter protein molecule by reactive oxygen species (ROS), but the mechanisms for increasing P-gp expression have not been elucidated. The transcription factors Nrf2 and HIF1 are supposed to be involved in this process [17, 18]. The aim of this study was to explore the mechanisms of P-gp regulation in OS.

## EXPERIMENTAL

**Cell culture**

In this study, we used the human colon adenocarcinoma Caco-2 cell line (Shared Research Facility “Collection of Vertebrate Cell Cultures”, Saint- Petersburg, Russia). The cells were cultured at 37°C and 5% CO_2_ in a WS-189C incubator (World Science, Korea) in a Dulbecco’s modified Eagle’s medium (DMEM) supplemented with high glucose (4,500 mg/L), L-glutamine (4 mM), 15% fetal bovine serum, 100 U/mL penicillin, and 100 μg/mL streptomycin (all reagents from Sigma-Aldrich, USA). The cells were seeded in six-well plates (Corning, USA); the well surface area was 9.6 cm^2^; the number of cells per well was 1.8–2.0 × 106; the working volume of the growth medium was 1.5 mL. The cells were cultured for 21 days, because such an amount of time was required for their spontaneous differentiation into enterocyte-like cells overexpressing P-gp [19].

In the study, the following experimental groups were formed:

1) Control (n = 3): cells incubated in the growth medium supplemented with an equivalent volume of water for injection (solvent of H_2_O_2_ and BSO);

2) P-gp induction under simulated OS conditions.

Exogenous OS was simulated by adding into the growth medium H_2_O_2_ at a concentration of 0.1, 0.5, and 1 μM for 24 h (5–50 × 10–17 mol/cell) and 10 μM for 72 h (5 × 10–15 mol/cell).

Endogenous OS was induced using an inhibitor of glutathione synthesis, DL-buthionine sulfoximine (BSO, γ-glutamylcysteine synthetase inhibitor) [20] at final concentrations of 10, 50, and 100 μM in the growth medium (5–50 × 10^–15^ mol/cell).

Pro-oxidant concentrations and exposure duration were chosen in accordance with the results of preliminary experiments on P-gp induction [17, 21].

3) OS inhibition: pro-oxidants and 1 mM glutathione were simultaneously added to the growth medium [22].

4) Evaluation of the role of the Nrf2-mediated mechanism in P-gp induction under OS: an inhibitor, N-(1,3-benzodioxol-5-ylmethyl)-5-(4-fluorophenyl)- thieno[2,3-d]pyrimidin-4-amine (AEM1, Sigma- Aldrich), at a concentration of 5 μM was added to the growth medium with cells 30 min before their exposure to H_2_O_2_/BSO [23].

5) Evaluation of the role of the HIF1-mediated mechanism in P-gp induction under OS: N,N’- (disulfanediylbis(ethane-2,1-diyl))bis(2,5-dichlorobenzenesulfonamide) (KC7F2, Sigma-Aldrich), at a concentration of 7.5 μM was added to the growth medium with cells 30 min before their exposure to H_2_O_2_/BSO [24].

6) Evaluation of the role of the CAR-mediated mechanism in P-gp induction under OS: an inhibitor, ethyl [5-[(diethylamino)acetyl]-10,11-dihydro-5H-dibenz[b,f]azepin-3-yl]carbamate (CINPA1, TOCRIS, UK), at a concentration of 10 μM was added to the growth medium with cells 30 min before their exposure to H_2_O_2_/BSO [25].

7) Evaluation of the role of the PXR-mediated mechanism in P-gp induction under OS: ketoconazole (Sigma-Aldrich) at a concentration of 10 μM was added to the growth medium with cells 30 min before their exposure to H_2_O_2_/BSO [26].

Each experiment was performed in triplicate. During 72-hour exposure, the growth medium containing a pro-oxidant and an inhibitor was changed every 24 h.

**Pro-oxidant-induced ROS overproduction was confirmed using fluorescent probes**

Cells were cultured in 24-well plates. After incubation with H_2_O_2_ for 3 h and BSO for 24 h at the tested concentrations, the level of intracellular ROS was assessed by staining the cells with MitoTracker Red CM-H2XRos (Invitrogen, USA). MitoTracker Red probes (non-fluorescent form) contain reduced dihydrorosamine that penetrates into living cells, binds to the thiol groups in mitochondria, and fluoresces upon ROS oxidation.

The cells were visualized using an Olympus CKX53 inverted microscope (Olympus, Japan), then detached from the wells and lysed using 0.2% Triton X-100 (Sigma-Aldrich; https://www.thermofisher.com/order/ catalog/product/M7513). The level of free radicals in the cell lysate was evaluated based on the fluorescence intensity (λ_ex_ = 579 nm, λem = 599 nm) using an RF-6000 spectrofluorometer (Shimadzu, Japan) and converted to the cell number using a Countess 3 Automated Cell Counter (USA).

In the remaining experiments, the cells were cultured in six-well plates.

**Preparation of complete cell lysates**

After the end of exposure to H_2_O_2_ and BSO, the cells were detached from the six-well plates using a trypsin–EDTA solution (0.25% trypsin and 0.2% EDTA, Sigma-Aldrich), washed three times with a phosphate buffer solution (BioRad, USA), and lysed in NP40 cell lysis buffer (ThermoFisher Scientific, USA) supplemented with a mixture of proteinase inhibitors (2 mM 4-(2 aminoethyl)benzenesulfonyl fluoride hydrochloride (AEBSF), 0.3 μM aprotinin, 130 μM bestatin, 1 mM EDTA, 14 μM trans-epoxysuccinyl-L-leucylamido(4-guanidino)butane (E-64), and 1 μM leupeptin, Sigma-Aldrich), 107 cells per 100 μL of the buffer, at +4°C and constant stirring for 30 min. The resulting lysate was centrifuged at 5,000 g (CM-50, Eppendorf, Germany). The supernatant was used in the biochemical analyses.

The protein content in the samples was evaluated by the Bradford method using a Pierce Coomassie plus (Bradford) assay kit (ThermoFisher, USA) [27].

**Evaluation of the relative P-gp content in Caco-2 cells by Western blot**

Supernatant proteins (20 μg) were subjected to electrophoresis using a 7.5% TGX Stain-Free FastCast acrylamide kit (BioRad, USA) in a Laemmli buffer system (BioRad). Samples were mixed with a Laemmli buffer containing 50 mM β-mercaptoethanol (Helicon, USA) at a 1:3 ratio and incubated at 70°C for 10 min. Electrophoresis was performed at 100 V for 90 min.

Proteins were transferred to a nitrocellulose membrane (Trans-Blot Turbo Mini-Size nitrocellulose, BioRad) using a Mini Trans-Blot Cell (BioRad) at 25 V and 1.3 A for 10 min.

The proteins on the membrane were blocked with a 1% casein blocker solution (BioRad) containing 0.1% Tween-20 (Sigma, Germany) at room temperature for 1 h.

The P-gp protein was detected using primary mouse monoclonal antibodies (P-glycoprotein antibody, MA5-13854, Invitrogen) at a dilution of 1:200 in a casein blocker solution (BioRad) at 37°C for 2 h. Primary antibodies were visualized by incubation with rabbit anti-mouse IgG (H + L) secondary antibodies, HRP (Invitrogen) (1:4,000 dilution) at room temperature for 1 h. Chemiluminescence was detected using a ChemiDoc XRS+ system (BioRad). Band intensities were evaluated densitometrically using the ImageLab software (BioRad).

The molecular weight of P-gp was confirmed by comparison with precision plus protein standards, dual color (BioRad).

The P-gp content was normalized to the housekeeping protein GAPDH content (primary antibodies GAPDH Loading Control Monoclonal Antibody (GA1R), DyLight 68 (Invitrogen), 1:1,000 dilution, secondary rabbit antibodies – Rabbit-anti-Mouse IgG (H + L) Secondary Antibody, HRP (Invitrogen, 1:4,000 dilution).

**Statistical analysis**

Data were analyzed using the GraphPad Prism 8 software. The results are presented as a mean ± standard deviation (M ± SD). The statistical sig nificance of the differences was assessed using an analysis of the variance (ANOVA); pairwise comparisons were performed using a Dunnett’s test. Differences were considered statistically significant at p < 0.05.

## RESULTS

**ROS production upon simulated oxidative stress**

**Fig. 1:**
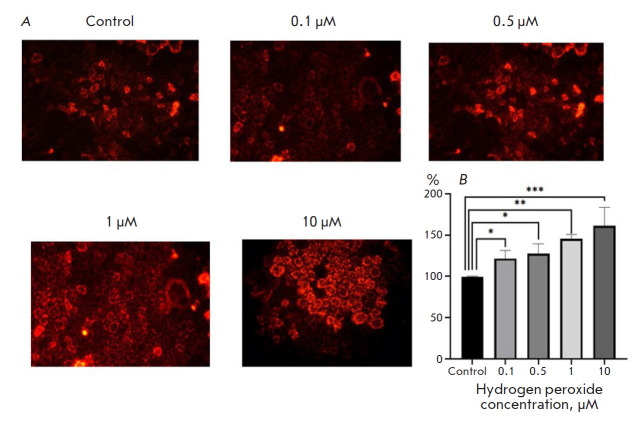
Hydrogen peroxide (H_2_O_2_)-induced changes in the ROS level in Caco-2 cells. (*A*) Staining with MitoTracker Red CM-H2XRos; magnification: 400×. (*B*) Fluorescence intensity in a cell lysate. *p ≤0.05; **p  < 0.01; ***p ≤0.001 compared with control (Dunnett’s test)

Exposure of Caco-2 cells to H_2_O_2_ at a concentration of 0.1, 0.5, 1.0, and 10 μM for 3 h resulted in an increase in the fluorescence intensity of the cells stained with MitoTracker Red CM-H2XRos by 21.5% (p = 0.05), 27.3% (p = 0.046), 45.4% (p = 0.004), and 61.1% (p = 0.001), respectively, compared with that in the control taken as 100% (Fig. 1).

**Fig. 2:**
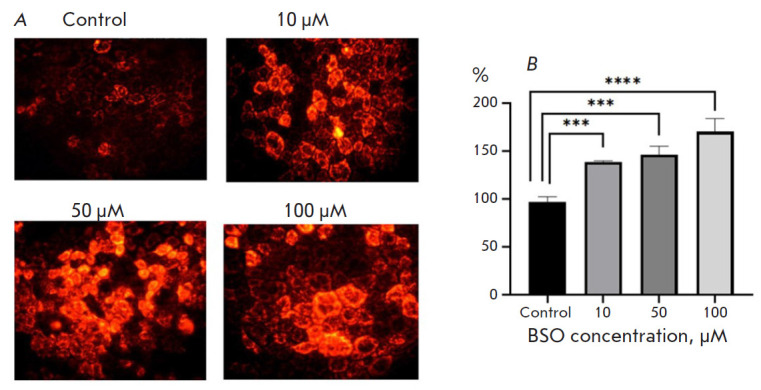
*D,L*-buthionine sulfoximine-induced changes in the ROS level in Caco-2 cells. (*A*) Staining with MitoTracker Red CM-H2XRos; magnification: 400×. (*B*) Fluorescence intensity in a cell lysate. ***p ≤0.001; ****p ≤0.0001 compared with control (Dunnett’s test)

Similarly, the fluorescence intensity of Caco-2 cells exposed to BSO at a concentration of 10, 50, and 100 μM for 24 h and stained with MitoTracker Red CM-H2XRos increased by 38.8% (p = 0.001), 46.5% (p = 0.0004), and 70.2% (p = 0.0001), respectively, compared with that in the control (Fig. 2).

These results indicate an increase in ROS production in the used experimental models.

**Changes in the relative P-gp content in Caco-2 cells under exogenous and endogenous oxidative stress**

**Fig. 3:**
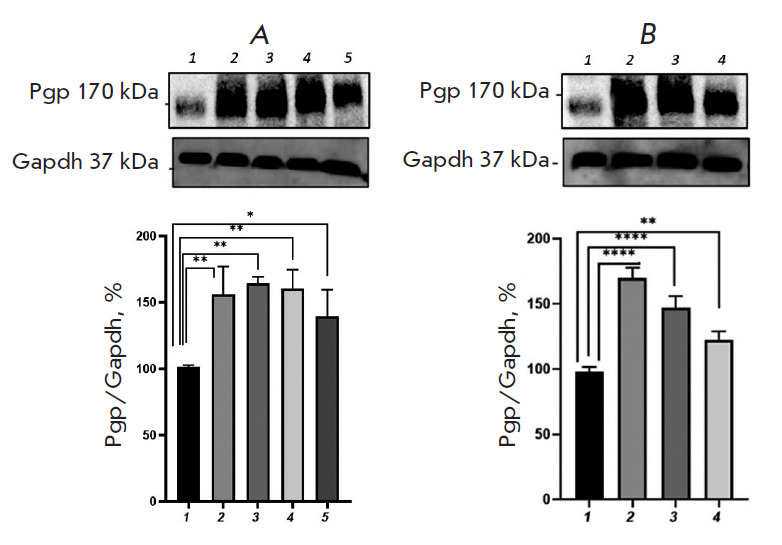
Relative P-glycoprotein content in Caco-2 cells exposed to H_2_O_2_ (*A*, exogenous oxidative stress) and *DL*-buthionine sulfoximine (*B*, endogenous oxidative stress). (*A*) 1 – control; 2, 3, 4, 5 – hydrogen peroxide at a concentration of 10 μM (72 h), 0.1, 0.5, and 1 μM (24 h), respectively. (*B*) 1 – control; 2, 3, 4 – *DL*-buthionine sulfoximine at concentrations of 10, 50, and 100 μM (24 h), respectively. *p  < 0.05; **p  < 0.01; ****p  < 0.0001, statistically significant differences from the control (Dunnett’s test)

Exposure to H_2_O_2_ (simulation of exogenous OS) at a concentration of 0.1, 0.5, and 1 μM for 24 h caused an increase in the P-gp content by 78.9% (p = 0.0013), 67.1% (p = 0.0019), and 44.6% (p = 0.029), respectively (Fig. 3A), compared with that in the control. An increase in the duration of the exposure to 72 h at an H_2_O_2_ concentration of 10 μM elevated the P-gp level by 68.9% (p = 0.0033), compared with that in the control (Fig. 3A).

**Fig. 4:**
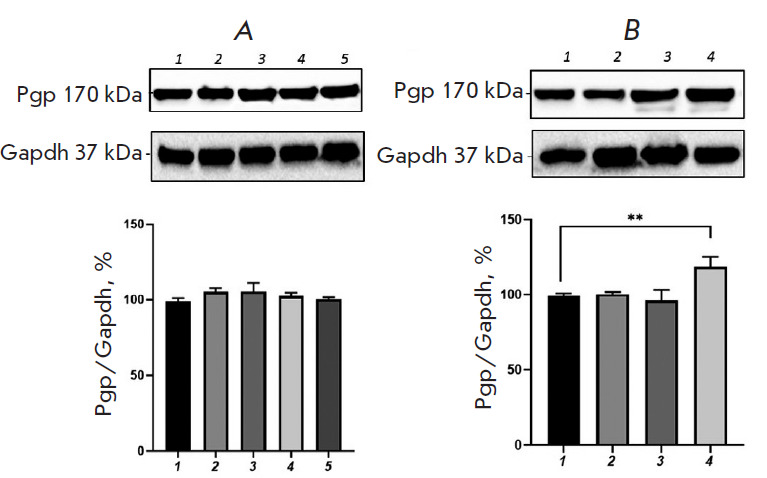
Relative P-glycoprotein content in Caco-2 cells exposed to H_2_O_2_ (*A*, exogenous oxidative stress) and *DL*-buthionine sulfoximine (*B*, endogenous oxidative stress) in combination with glutathione (1 mM). (*A*) 1 – control; 2, 3, 4, 5 – hydrogen peroxide at a concentration of 10 μM (72 h), 0.1, 0.5, and 1 μM (24 h), respectively. (*B*) 1 – control; 2, 3, 4 – *DL*-buthionine sulfoximine at concentrations of 10, 50, and 100 μM (24 h), respectively. **p  < 0.01, statistically significant differences from the control (Dunnett’s test)

Incubation of Caco-2 cells with BSO (simulation of endogenous stress) at a concentration of 10, 50, and 100 μM for 24 h resulted in an increase in the relative P-gp content by 71.6% (p < 0.0001), 51.6% (p < 0.0001), and 25.4% (p = 0.007), respectively (Fig. 3B).

Upon increasing exposure to 72 h, the effect of BSO was eliminated and the P-gp level did not differ significantly from that in the control.

The addition of GSH at a concentration of 1 mM to the growth medium containing H_2_O_2_ at all concentrations and for all incubation periods prevented any increase in the P-gp content; its level did not differ significantly from that in the control (Fig. 4A).

Upon combined use of 1 mM glutathione and 100 μM BSO and incubation for 24 h, the relative P-gp content increased by 19.7% (p = 0.003) compared with that in the control; however, this increase was less pronounced than that when the pro-oxidant was used alone. In this case, GSH prevented any increase in the P-gp level caused by exposure to BSO at the lower concentrations of 10 and 50 μM for 24 h (Fig. 4B).

Therefore, exposure of Caco-2 cells to H_2_O_2_ and BSO (simulation of exogenous and endogenous OS) leads to an increase in the P-gp level, and the use of the endogenous antioxidant glutathione eliminates this induction, except for the exposure to BSO (100 μM) for 24 h.

**Investigation of the mechanisms increasing the P-gp level in the presence of hydrogen peroxide and DL-buthionine sulfoximine**

The mechanisms associated with an increased P-gp level under exogenous and endogenous OS were studied using the transcription factor inhibitors AEM1 Nrf2), KC7F2 (HIF1α), CINPA1 (CAR), and ketoconazole (PXR), which stimulate the expression of the MDR1 gene encoding P-gp.

**Fig. 5:**
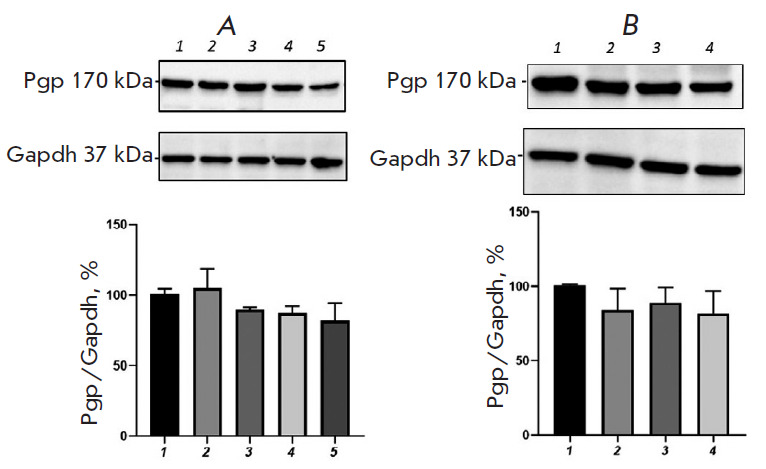
Relative P-glycoprotein content in Caco-2 cells exposed simultaneously to an Nrf2 inhibitor (AEM1, 5 µM) and H_2_O_2_ (*A*) or *DL*-buthionine sulfoximine (*B*). (*A*) 1 – control; 2, 3, 4, 5 – hydrogen peroxide at a concentration of 10 μM (72 h), 0.1, 0.5, and 1 μM (24 h), respectively. (*B*) 1 – control; 2, 3, 4 – *DL*-buthionine sulfoximine at concentrations of 10, 50, and 100 µM (24 h), respectively

Co-incubation of the Nrf2 inhibitor AEM1 (5 μM) with H_2_O_2_ (all concentrations and exposure times) prevented any increase in the relative P-gp content; its level did not differ significantly from that in the control (Fig. 5A).

The addition of AEM1, in combination with BSO (10, 50, and 100 μM), and incubation for 24 h also prevented any increase in the relative P-gp content; the transporter protein level did not differ from that in the control) (Fig. 5B).

**Fig. 6:**
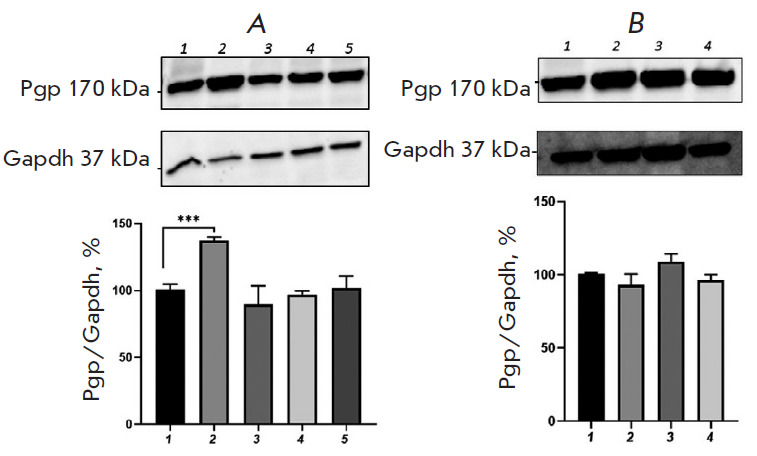
Relative P-glycoprotein content in Caco-2 cells exposed simultaneously to an HIF1α inhibitor (KC7F2, 7.5 µM) and H_2_O_2_ (*A*) or *DL*-buthionine sulfoximine (*B*). (*A*) 1 – control; 2, 3, 4, 5 – hydrogen peroxide at a concentration of 10 μM (72 h), 0.1, 0.5, and 1 μM (24 h), respectively. (*B*) 1 – control; 2, 3, 4 – *DL*-buthionine sulfoximine at concentrations of 10, 50, and 100 μM (24 h), respectively. ***p  < 0.001, statistically significant differences from control (Dunnett’s test)

The HIF1α inhibitor KC7F2 (7.5 μM) prevented any increase in the transporter level in the presence of H_2_O_2_ (24 h, all concentrations); the relative P-gp content did not differ significantly from that in the control. Incubation with KC7F2 for 72 h did not significantly affect the relative P-gp content; its content increased by 37% relative to that in the control (p = 0.0004) (Fig. 6A).

The addition of KC7F2 to cells incubated with BSO (10, 50, and 100 μM) also led to a normalization of the relative P-gp content; its level did not differ significantly from that in the control (Fig. 6B).

**Fig. 7:**
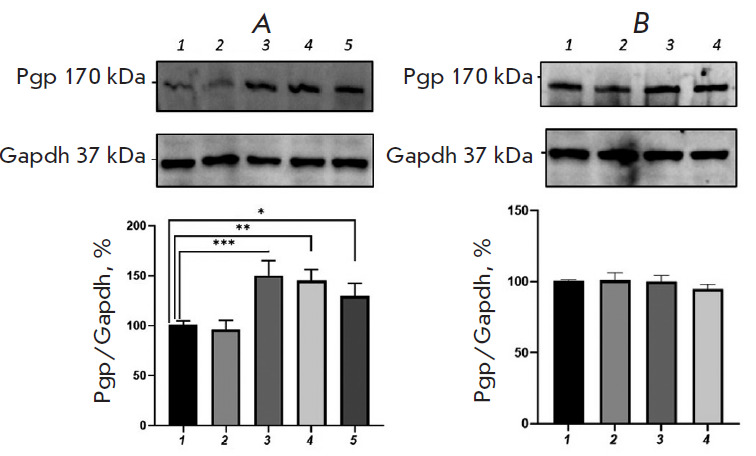
Relative P-glycoprotein content in Caco-2 cells exposed simultaneously to a CAR inhibitor (CINPA1, 5 μM) and H_2_O_2_ (*A*) or *DL*-buthionine sulfoximine (*B*). (*A*) 1 – control; 2, 3, 4, 5 – hydrogen peroxide at a concentration of 10 μM (72 h), 0.1, 0.5, and 1 μM (24 h), respectively. (*B*) 1 – control; 2, 3, 4 – *DL*-buthionine sulfoximine at concentrations of 10, 50, and 100 μM (24 h), respectively. *p  < 0.05; **p  < 0.01; ***p  < 0.001, statistically significant differences from the control (Dunnett’s test)

The addition of the CAR inhibitor CINPA1 (5 μM) to the cells incubated with 0.1, 0.5, and 1 μM H_2_O_2_ for 24 h did not suppress the pro-oxidant effect; the relative P-gp content increased by 51.5% (p = 0.0008), 46.5% (p = 0.0019), and 31.3% (p = 0.02), respectively, compared with that in the control (Fig. 7A).

However, prolonged incubation (72 h) with CINPA1 prevented any increase in the P-gp content under the action of 10 μM H_2_O_2_ (Fig. 6A).

CINPA1 combined with BSO (10, 50, and 100 μM) prevented any increase in the relative P-gp content; the transporter protein level did not differ significantly from that in control (Fig. 7B).

**Fig. 8:**
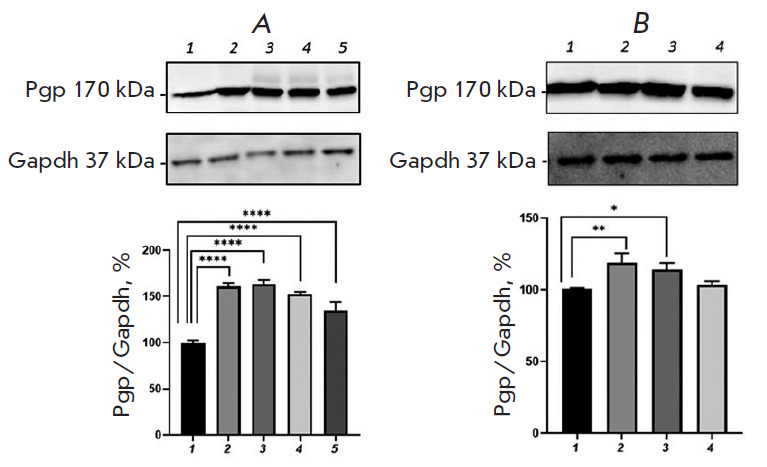
Relative P-glycoprotein content in Caco-2 cells exposed simultaneously to a PXR inhibitor (ketoconazole, 10 µM) and H_2_O_2_ (*A*) or *DL*-buthionine sulfoximine (*B*). (*A*) 1 – control; 2, 3, 4, 5 – hydrogen peroxide at a concentration of 10 μM (72 h), 0.1, 0.5, and 1 μM (24 h), respectively. (*B*) 1 – control; 2, 3, 4 – *DL*-buthionine sulfoximine at concentrations of 10, 50, and 100 μM (24 h), respectively. *p  < 0.05; **p  < 0.01; ****p  < 0.0001, statistically significant differences from the control (Dunnett’s test)

The PXR inhibitor ketoconazole (10 μM) with H_2_O_2_ did not suppress the effect of the oxidative stress inducer. The relative P-gp content increased by 64.6, 53.5, and 36.4% upon exposure to H_2_O_2_ (0.1, 0.5, and 1 μM, 24 h) and by 62.6% upon exposure to H_2_O_2_ (10 μM, 72 h), p < 0.0001 in each series of experiments, Fig. 8A.

At the same time, ketoconazole prevented any increase in the P-gp content under the action of BSO at a concentration of 100 μM and did not affect the effect of the pro-oxidant at a concentration of 10 and 50 μM; the P-gp level increased by 18.8% (p = 0.0027) and 14.1% (p = 0.015), respectively, compared with that in the control (Fig. 8B).

Therefore, P-gp is regulated mainly through the Nrf2-Keap1 signaling pathway under exogenous OS (H_2_O_2_), while all of the studied transcription factors are involved in the regulation of P-gp under endogenous OS (BSO).

## DISCUSSION

Oxidative stress is a redox-dependent process associated with many pathologies of various origins. The cause of OS can be exogenous (exposure to a pro-oxidant) and/or endogenous (suppression of the intracellular antioxidant defense) [28].

In the present study, exogenous OS was modeled by exposure of Caco-2 cells to hydrogen peroxide. H_2_O_2_ is able to penetrate through cell membranes. In cells, H_2_O_2_ interacts with metals of variable valency (Fe^2+^ or Cu^+^) in the Fenton and Haber–Weiss reactions to form highly toxic, oxygen-containing free radicals (hydroxyl radical •OH and superoxide anion O_2_•-), which can cause oxidative damage to cell biomacromolecules [29]. Given the micromolar range of the H_2_O_2_ concentrations used in the study and the rapid elimination of H_2_O_2_ by the cells [30], the revealed changes in the P-gp content are most likely due to the signal cascades triggered by the pro-oxidant.

Endogenous OS was induced by incubation of the cells with BSO that inhibits the γ-glutamylcysteine synthetase (γ-GCS) enzyme that plays a key role in the synthesis and maintenance of the cellular glutathione level. Glutathione (GSH) is a thiol-containing tripeptide that exhibits antioxidant activity and is necessary for the functioning of antioxidant enzymes (glutathione peroxidase, glutathione S-transferase). A decrease in the level of endogenous glutathione reduces the endogenous antioxidant system capacity, which provokes OS development [31].

The dynamics of OS development was confirmed in this study by ROS detection based on the fluorescence intensity of MitoTracker Red CM-H2XRos. Exposure to H_2_O_2_ caused an increase in ROS in 3 h, while exposure to BSO increased the concentration of ROS in 24 h, after the endogenous glutathione pool had been depleted.

The development of both exogenous and endogenous OS led to an increase in the relative P-gp content. However, addition of the antioxidant glutathione and used pro-oxidants to the cells prevented P-gp induction by H_2_O_2_ and reduced (100 μM) or suppressed (10 and 50 μM) P-gp induction by BSO.

Partial suppression of P-gp induction by glutathione may be associated with the fact that BSO, being a xenobiotic, can increase the P-gp content via stimulation of the MDR1 gene expression.

Currently, several mechanisms of P-gp regulation are known, the main one being alteration of the expression of the MDR1 gene that encodes the transporter protein [31].

In this study, we evaluated the role of the Nrf2, HIF1a, CAR, and PXR transcription factors, which are activated under oxidative stress [17, 33, 34, 35] and may hypothetically elevate P-gp expression.

The Nrf2 signaling pathway is considered to be the main mechanism that regulates the antioxidant defense of cells in OS. Under physiological conditions, the nuclear transcription factor Nrf2 is involved in the Keap1-Nrf2-Cullin-3 complex, which ensures that it remains in the cytosol and blocks its specific activity. Nrf2 is a redox-sensitive transcription factor; oxidation of the SH-groups in Keap1 leads to the activation of the factor, its translocation to the nucleus, and alteration of biological effects – induction of antioxidant enzymes [36].

The hypoxia-inducible factor (HIF1) is a transcription factor that plays a key role in a cell’s adaptation to decreasing oxygen levels in tissues [37]. HIF1 is a heterodimer composed of two protein subunits, HIF1α and HIF1β. The functional status of HIF1 is controlled by the expression and activity of its α-subunit that is regulated on several levels: transcription, translation, post-translational changes, and translocation to the nucleus [38]. Under normoxic conditions, oxygen-dependent proline hydroxylases modify proline in HIF1α. Under OS, proline hydroxylases are inactive; in these conditions, the α- and β-subunits are able to bind to each other, penetrate into the nucleus, and activate the expression of the target genes.

The constitutive androstane receptor (CAR; nuclear receptor subfamily 1 group I member 3, NR1I3) and pregnane X receptor (PXR; steroid and xenobiotic receptor SXR; nuclear receptor subfamily 1 group I member 2, NR112) are members of the nuclear receptor superfamily that is comprised mainly of transcription factors [39].

These receptors are localized mainly in the liver and intestines, where they regulate the expression of phase I biotransformation enzymes, such as cytochrome P450 isoenzymes CYP3A and CYP2B, and transporter proteins: in particular P-gp.

The relative contents of CAR and PXR increase under OS conditions in response to the accumulation of peroxidation products [34, 35].

The role of Nrf2 in P-gp regulation was assessed using AEM1 (ARE expression modulator 1) that blocks the interaction between Nfr2 and ARE (antioxidant respons(iv)e element HIF t) and suppresses the expression of the genes controlled by this transcription factor. AEM1 was found to block the ability of H_2_O_2_ and BSO (at all applied concentrations and exposure times) to induce P-gp.

Therefore, Nrf2 is involved in P-gp regulation under both exogenous and endogenous OS.

The HIF1α inhibitor KC7F2 (controls the biological activity of HIF1α) is a symmetrical compound that selectively inhibits the cellular synthesis of HIF1α, but not HIF1β, without affecting HIF1α mRNA transcription or HIF1α protein stability. In cells exposed to H_2_O_2_ and BSO for 24 h, KC7F2 normalized the P-gp level (prevented pro-oxidant-mediated induction); during exposure, in combination with H_2_O_2_ for 72 h, KC7F2 had no significant effect (the relative P-gp content increased under exposure to hydrogen peroxide).

Thus, there are two transcription factors, Nrf2 and HIF1, which are involved in P-gp regulation under both endogenous and exogenous OS. Both factors may be capable of binding to the promoter of the MDR1 gene, which encodes P-gp, and increasing its expression. We have previously shown that Nrf2 causes an increase in HIF1α expression during OS [33]; i.e., Nrf2 can act through HIF1α. Because Nrf2 inhibition, in contrast to HIF1α, prevented P-gp induction in all during incubation for 24 and 72 h under exogenous and endogenous OS, the two described mechanisms apparently function in tandem in the cell.

In the present study, we used CINPA1 (CAR inhibitor, not PXR activator 1) as a CAR inhibitor; CINPA1 interacts with and blocks the CAR ligand-binding domain and inhibits its binding to co-activators [40]. For PXR inhibition, we used an antifungal agent from the azole group, ketoconazole, which binds to the AF-2 (activation function) region of the N-terminal ligand-binding domain of PXR and, thus, suppresses its activation [41].

CINPA1 did not suppress P-gp induction by H_2_O_2_ upon 24-hour incubation, and it prevented any increase in the P-gp level upon 72-hour exposure. H_2_O_2_ was shown to induce CAR [34] that, in turn, apparently increases P-gp expression upon 72-hour exposure.

The combined use of BSO and CINPA1 prevented an increase in the relative P-gp content; the transporter protein level did not differ significantly from that in the control.

The PXR inhibitor ketoconazole, applied together with H_2_O_2_, did not suppress the effect of the OS inducer. However, ketoconazole completely prevented an increase in the P-gp level under the action of BSO at a concentration of 100 μM and, partially, at concentrations of 10 and 50 μM. CAR and PXR are the main intracellular xenosensory receptors; i.e., they interact with xenobiotics and trigger an intracellular response to neutralize and eliminate the xenobiotics.

BSO, being a xenobiotic, may be suggested to independently activate CAR and PXR, and they, in turn, increase P-gp expression. The persistence of an ele vated P-gp level during the combined use of BSO and glutathione, which was revealed in the present study, supports this suggestion.

It is interesting to note that upon simulation of both exogenous and endogenous oxidative stress, despite the simultaneous involvement of different transcription factors in P-gp induction, inhibition of only one of them led to the suppression of any increase in the P-gp content, which indicates that several mechanisms should act simultaneously to induce P-gp in certain situations.

## CONCLUSION

In conclusion, an increase in the P-gp content under exogenous OS induced by the incubation of Caco-2 cells with H_2_O_2_ is primarily mediated by the Nrf2- Keap1 signaling pathway that is involved in the regulation of the transporter protein at exposure durations of 24 and 72 h. The transcription factors HIF and CAR are involved in the P-gp regulation upon 24-hour and 72-hour exposure to H_2_O_2_, respectively. Apparently, PXR does not significantly affect the regulation of the transporter protein in this OS model.

Simulation of endogenous OS in Caco-2 cells using the glutathione synthesis inhibitor BSO revealed that all tested transcription factors and signaling pathways are involved in the P-gp induction. Most likely, this is due to the bimodal effect of BSO on P-gp. On the one hand, BSO induces OS; on the other, being a xenobiotic, BSO is able to stimulate PXR and CAR.

## Abbreviations

ROS: reactive oxygen species
BSO: DL-buthionine sulfoximine
OS: oxidative stress
CAR: constitutive androstane receptor
HIF1α: hypoxia-inducible factor 1a
Nrf2: nuclear factor erythroid 2-related factor 2
PXR: pregnane X receptor
Pgp: P-glycoprotein
